# Health care and harm reduction provider perspectives on treating older adults who use non-medical opioids: a qualitative study set in Chicago

**DOI:** 10.1186/s12913-023-09843-4

**Published:** 2023-08-19

**Authors:** Maryann Mason, Lori Ann Post, Rahul Aggarwal

**Affiliations:** 1 Feinberg School of Medicine, Northwestern University, Chicago, 60611 USA; 2 Buehler Center for Health Policy and Economics, Northwestern University, 420 E. Superior St., Rubloff Building 9th floor, Room 939, Chicago, IL 60611 USA; 3 Department of Emergency Medicine, The Buehler Center for Health Policy & Economics, Institute for Global Health, Institute for Public Health and Medicine, Northwestern Feinberg School of Medicine, 9-915 Rubloff Building, 420 E. Superior St, Chicago, Il 60611 USA; 4 Weinberg School of Arts and Sciences, Northwestern University, 1918 Sheridan Rd, Evanston, IL 60208 USA

**Keywords:** Older adults, Non-medical opioid use; Harm reduction, Health care, Substance use treatment, Substance use screening, OUD

## Abstract

**Background:**

Opioid overdose death rates are increasing for adults aged 55 and older, with especially high rates in large urban areas. In parallel, admissions to treatment programs for older adults using illicit substances are increasing as well. Despite these trends, there is a lack of information about older adults who use non-medical opioids (NMO) and even less knowledge about their health and service encounters. Conducted in Chicago, Illinois, this qualitative study explores the perspectives of health care and harm reduction service providers who work with older adults using non-medical opioids.

**Methods:**

The study used snowball sampling to locate participants with expertise in working with older adults who use non-medical opioids. In total, we conducted 26 semi-structured interviews from September 2021-August 2022. We explored questions regarding participants’ perceptions of older adult opioid use patterns, comorbidities, and involvement in harm reduction outreach and opioid use disorder treatment.

**Results:**

Many of the providers we interviewed consider older adults who use NMO as a distinct population that employ unique use behaviors with the intent to protect them from opioid overdose. However, these same unique behaviors may potentiate their risk for overdose in today’s climate. Providers report initial encounters that are not care seeking for opioid use and primarily oriented around health conditions. Older adults who use non-medical opioids are seen as complex patients due to the need to diagnostically untangle symptoms of substance use from co-morbidities and conditions associated with aging.

Treatment for this population is also viewed as complicated due to the interactions between aging, comorbidities, and substance use. Providers also noted that older adults who use NMO have use behaviors that make them less visible to outreach and treatment service providers, potentially putting them at increased risk for overdose and health conditions associated with opioid use.

**Conclusions:**

Findings from this study are intended to inform future research on care provision for older adults who use non-medical opioids and may be especially applicable to large urban reas with histories of opioid use dating back to earlier drug epidemics of the 1970s, 1980s, and 1990s.

## Background

The US is currently in the midst of its fifth opioid crisis, the first dating back to the Civil War. One of the first documented US outbreaks of mass addiction involved morphine, used to treat soldier combat wounds during the Civil War [1]. Up until the 1920s, opium was sold in store fronts without prescriptions or could be smoked in opium dens in major urban centers [2]. Heroin and cocaine epidemics of the 1970’s,1980’s and early 1990’s plagued urban and minoritized communities in the US [3]. The current opioid crisis is now entering its third decade and is widely acknowledged to have begun with the excessive prescription of opioids in the early 2000s.

More than half (69%) of older adults aged 55 years and older who initiated treatment for heroin use between 2008 and 2017, began their use before the age of 30 [4]. Thus, it is likely that a significant proportion of today’s older adults who use non-medical opioids (NMO) began their use during these prior epidemics, or in the early days of the current epidemic. Today’s older adults have continued their non-medical drug use into their later years at higher rates than any prior generation [5, 6] and older adult admissions for substance use treatment have increased rapidly in recent years [6, 7]. In 2021, the National Survey on Drug estimated 13.5% of adults aged 50 years and older used an illicit substance in the last year [7].

Currently, opioid overdose deaths (OODs) are increasing among older adults (55 + years). The OOD rate per 100,000 persons aged 55 years and older increased from 0.9 in 1999 to 13.6 in 2020. Non-Hispanic Black males have OOD rates four times greater than the overall OOD rate for the same aged persons [8–10]. Beginning in 2016, older adult OOD rates in large central metropolitan grew faster and outpaced OOD rates in other areas [11].

Health care and harm reduction encounters can play a key role in prevention of OOD, providing key touchpoints for screening, treatment initiation, and harm reduction outreach [12]. However, despite increasing rates of older adult illicit opioid use, opioid use disorder (OUD), and OOD, there remains a dearth of information on older adult non-medical opioid use and help seeking behaviors. We found few current (published within the last five years) studies on older adult use behaviors such as methods of ingestion, social networks among older adults who use substances, and substances and combinations of substances used. We did find limited data on types of opioids used and motivations for using in this population. Using data from a 2015 to 2017 national drug use survey, Schuler et al. report that older adults who “misuse” prescription opioids are more likely to have a medical source for their opioids, compared to younger people, and cite pain relief as their primary motivator for substance use [13]. Another study identified the role of social and familial networks in the initiation of substance use among incarcerated older adults with a history of drug use [14]. We could not locate studies on harm reduction behaviors or drug use management strategies among older adults in the US who use substances. Information about health care encounters is also lacking [15]. Yet, information on these encounters is likely to be especially informative in urban areas like Chicago, which experienced drug epidemics in the 1970s, 1980s, and early 1990s and is highly affected by today’s opioid crisis. To investigate, we conducted a qualitative study with health care and harm reduction providers working with older adults who use non-medical opioids in Chicago. Because of the limited current literature on older adult use behaviors, we also incorporated questions for health care providers on their perceptions of older adult non-medical opioid (NMO) use, including methods of ingestion, social networks, and specific substances used to provide additional context on the issue. This study focuses on provider perceptions of this population, their perceptions of health needs, and presentation to care and risks related to their NMO use. Our goal is to inform future research and work with this population.

## Methods

### Human subject protections

This study (#15,454) was deemed exempt by the Northwestern University Institutional Review Board under category 2(i).

### Study design

This is a qualitative study designed to surface major themes among the perspectives of health care, harm reduction, and substance use disorder treatment providers who care for older adults using NMO in Chicago. A semi-structured interview protocol was developed based on a review of literature and bounded by the purpose of the study to gain perspectives on health and harm reduction seeking behaviors of older adults who use NMO. We set the age of “older adult” at 55 years and older based on increasing similarity of these age groups—more older adults are now working into older ages [16], health care access is similar with Medicaid expansion [17]; and drug dependence has been shown to cause premature aging, so older adults who are chronologically 55 to 64 years old may behave physiologically older than their age [18].

### Sampling

We used snowball sampling to locate care providers with experience working with older adults who use NMO. MM participates in overdose prevention efforts throughout the region and has knowledge regarding health care providers and sites working with older adults using NMO. Selection of initial providers was based on this contextual knowledge. We started with three providers known in the overdose prevention community for their expertise with older adults using NMO. Before the conclusion of every interview, we asked for names of additional care providers who could lend relevant insight.

### Participants

We interviewed 26 participants between September 2021-August 2022. Participants included physicians, nurses, social workers, harm reduction street outreach program workers and managers, and substance use disorder treatment providers working with older adults who use NMO in Chicago. The demographic breakdown of participants is as follows: 48.1% Male; 51.9% Female; 66.7% Non-Hispanic White, 11.1% Non-Hispanic Black and 22.2% identified as another race or ethnicity. The “other” category was developed to encompass less prevalent ethnic backgrounds among participants and to avoid the possibility of identification of individual participants.

### Setting

All participants worked in Chicago, Illinois. Those working in outreach and harm reduction were in areas with long-standing drug overdose problems. Cook County, Illinois, where Chicago is located, is a large, diverse county in Northeast Illinois. Cook County consistently had the highest number of annual OODs of any county in the US between 1999–2020 [8]. In 2019, OOD rates for those 55 + years were among the highest of all age groups in Cook County, Illinois [8]. In 2020, about a third (32.1%/534) of Cook County’s 1,662 OODs were among those 55 years and older. The age adjusted OOD rate for persons 55 years and older was 35.4 deaths per 100,000 persons aged 55 years and older, compared to 30.5 for persons less than 55 years. This setting provides a rich context in which to study the health and help seeking of older adults who use NMOs.

### Data collection

Interviews were conducted via Zoom Video Communications (San Jose, CA) and lasted between 18 and 54.5 min; average interview length was 33 min. In interviews, we explored participants’ perceptions of older adult NMO use patterns, co-morbidities, heath care, harm reduction outreach and opioid use disorder treatment. We encouraged participants to reflect specifically on the older adults they encountered in their respective roles.

### Data management

We recorded interviews using the Zoom Video Communications software with transcripts automatically generated. We de-identified transcripts by assigning a participant ID and removing names. We also removed references to health systems and exact locations to avoid identification through contextual clues. We cleaned transcripts to resolve transcription errors by reviewing the recording and editing the transcript when necessary.

### Data analysis

Each transcript was inductively analyzed, using a Directed Content Analysis Approach [19] in which we identified patterns in the data through an iterative process of data familiarization, coding, theme development and refinement. We used dedoose (www.dedoose.com), qualitative analysis software to code and analyze the interview transcripts. First, coder one (MM) read five randomly selected interview transcripts to identify initial coding themes. We revised codes based on discussion and the revised codes became the initial coding buckets. MM and RA each coded the same five transcripts using the revised coding buckets and compared outcomes. Our initial rates of agreement per code ranged from 36 to 80%. We calculated rates of agreement as number of coding applications in agreement for code X divided by the total number of segments coded with code X multiplied by 100. So, for example: (3/5)*100 converts to a rate of agreement of 60%. To address coding disagreement, MM and RA reviewed coded segments and referenced data to revise coding buckets. In doing so we combined some codes, split some into multiple codes, and more clearly defined rules for applying codes where there was ambiguity. Once, we refined the codebook, both coders once again independently coded all transcripts, meeting to discuss and refine coding as needed. After revisions, rates of agreement ranged between 95 and 100 percent for each of the 22 codes.

As a final check on our analyses two participants reviewed the draft manuscript to look for areas of disagreement, missing content and/or misrepresentation [20]. Both reviewers had minor feedback and thematic analysis findings were unchanged after the review.

## Results

### Contexts in which study participants interact with older adults using NMO

Of the 26 providers interviewed, one third were providing primary, hospitalist, and rehabilitative care (MDs and nurses), one third were MDs in a specialty area (pain management, emergency medicine), and one third were affiliated with harm reduction outreach, research, and mobile care units. Participants involved in harm reduction and street outreach worked in areas with long-standing drug overdose problems.

Participants provided care to older adults who use NMOs in clinics, hospitals, emergency rooms, rehabilitation facilities, mobile clinics, and in community contexts (e.g., street outreach). Harm reduction specialists worked in mobile service provider settings (vans) or directly on the street. By far, participants working with patients/clients in primary care and outreach settings reported more exposure to, experience with, and longitudinal relationships with older adults using NMO. The emergency medicine, hospitalists, and pain management physician participants had shorter episodic encounters with older adults who use NMOs and they usually presented with acute injuries or post-surgical care needs. Emergency department providers also saw older adults who use NMO presenting with opioid overdose.

### Older adults who use NMOs

Participants described older adults who used NMO in their care as majority African American or Black, mostly identifying as males, living in Chicago, with a decades-long opioid use history, and primarily using heroin or exhibiting the intention to use heroin, over prescription opioid pills.

Participants described distinct characteristics of older adult NMO use including preference for insufflation (snorting) over injection, preference for powders (mostly heroin) over prescription pills, preference for intermittent use as opposed to daily use and ingesting smaller quantities of opioids (1–2 bags vs 6–10 bags a day). Table 1 highlights quotes on the distinguishing features of older adults who use NMOs.

**Table 1:** Distinguishing features of older adults who use non-medical opioid

		Participant #
Race	*Majority of our older patients identify as Black and with our younger patients it’s a mix, but it’s more white patients who are younger that we are seeing. We see a lot of young people come into the city to buy drugs and then we see them at the syringe exchange. More of our older patients live in the inner communities in Chicago. Our younger patients who are injecting tend to identify as white*	22
Sex	*Around two-thirds of our patients [who use non-medical opioids] are older adults in their 50’s, 60’s, and 70’s. Most of our older adults are black and usually men*	18
Use behaviors		
Use powder vs. Pills	*I think heroin and fentanyl is a lot more accessible for most folks [older adults] as opposed to prescription. A prescription can only be obtained if you know of someone who has a prescription…Very often it’s powder, which is very different from younger white users I interviewed who started on a trajectory of using their parents’ medications, or they have behavioral issues so they got prescribed medications, or they’re part of a high school group in the summer where they might have had access to someone’s grandma’s pills. I think with this younger group, there is a lot of early exposure to pharmaceuticals but with older folks who I’ve interviewed, who tend to be African American, it tends to be street powders*	3
	*Pills are really expensive, so they (older adults) don’t buy that. It’s mainly heroin, and by heroin, I mean fentanyl. It’s mainly fentanyl that’s mixed with baby powder or whatever they cut it with*	20
Method of ingestion: snorting	*When we’re seeing older groups, they tend to be African American and don’t inject. Most of them tend to be people who snort opioids if they’re using opioids*	3
	*As far as IV vs. intranasal, a lot of people have a perception that IV use is serious use. But I’m not sure of a medical reason as to why people [older] are using intranasal*	18
	*People who are injecting are mostly younger. Because I provide wound care…I intend to attract a lot of the younger crowd. I also see a fair amount of folks who are insufflating as well and those tend to be older and African American*	17
	*As far as injection vs insufflation, you will have around 25% injection and 75% insufflation, split with the majority of injectors being younger than 40*	17
	*Particularly with African American individuals, they’re often in a treatment program and have gone through a period of abstinence from injection and they’re using the methadone and have done that practice for a while. They have resumed use. This may be because of the stigma around injecting, and they want to keep people unaware of their substance use. This is a more clandestine way to use. As a group, African Americans—you don’t see a lot injecting. It’s not a popular route of administration*	3
Long term use	*Most of my older adults over 50 have been using for decades and it’s been heroin. Now it’s fentanyl and it’s a 50/50 split between those who think they have heroin and its actually fentanyl and those who know they are getting fentanyl*	17
	*Majority of them [older adults] have been using for a long, long, long, long time. Also, they don’t have many overdoses. We have some patients who have been using 30* + *years*	22
	*The population we see are people who have been using for decades. It’s not someone who just got into a car accident last month and started using pills and maybe now has developed OUD. Given that its long-term use, they are less likely to use pills*	18
	*Oftentimes, people will have been using for decades, but that decades long use is intermittent. People will kick it back up again and in older adults I see that this happens usually after a traumatic event like loss of a parent, friend, spouse*	18
	*These are patients who, on average, have been using for decades as opposed to years. This may be very different than the perception of who the opioid epidemic has been affecting for the last 20 years*	18
	*All patients start with ‘stop opioids altogether’ since that’s what they want us to hear as providers. You can’t stop 30 years of use with one dose of suboxone. It’s a great long-term goal, but what do you want to do for next week?*	20
Intermittent use/use of lessor amounts	*They [older adults] tend not to use that much, maybe 1–2 bags a day. Younger adults maybe use 6–10. It’s more chronic, long-term use in older adults. I’m not sure if this is because these are the ones who survived*	20
	*They [older adults] didn’t have as many overdoses because they may be used once a day. A lot of the older adults are more functional addicts. They tend to work and take care of their kids. I don’t see this as much in younger adults, they tend to be fully into it and their lives are usually disoriented*	20
	*They [older adults] maybe use once or twice, or a couple times a week. Overall, majority of them use a smaller amount of heroin and fentanyl. Majority of them have been using for a long, long, long, long time. Also, they don’t have many overdoses. We have some patients who have been using 30* + *years*	22
	*It would be rare to encounter a 65-year-old patient who has been injecting 10 bags of heroin a day for a long, long, long time. Snorting a bag or two a day, a couple times a week*	22
	*They started usually before the '90 s oxycontin scare and they have always been using heroin. The biggest issue with them is that they are not using 10 bags a day, they are using probably a couple bags a day or every other day. They may be using it just to feel normal, like everyone else, or they may have painful comorbidities*	5

We present findings pertinent to care provider perceptions of older adults who use NMOs as patients/clients, challenges to providing care for this population, and ways in which aging interacts with NMO use. Findings are summarized as themes below. Table 2 includes quotes representative of themes.

**Table 2:** Representative thematic quotes

Theme	Representative quote	Participant #
*Care seeking*	*Oftentimes when I am seeing older patients, the OUD is not at the forefront of their visit. They may come for something else entirely*	*18*
	*We see them more in the outreach work in the field, rather than our field sites. Or if they come to our field sites, they’re usually using other services, such as HIV testing or other care*	*3*
*Complicated patients*	*The first thing I notice about older adults who are misusing opioids is that they are very, very sick. In addition to addiction and OUD, they frequently have chronic medical issues—cardiac issues, respiratory, neurologic. They are complicated*	*18*
	*They may need a knee replacement. However, you can’t find a surgeon to operate on them since a surgeon won’t operate on someone with OUD. This creates a cycle where the surgeon will say they won’t operate on someone with too many opioids, but the patient can’t get off of opioids because they are in too much pain. It’s a really difficult cycle to get out of. It’s really hard to ask patients to get off of opioids because they will be in incredible amounts of pain all the time*	*5*
	*If people have severe OUD that is not controlled, it becomes very difficult to manage any other comorbidity. Addiction introduces a level of chaos into people’s life and that makes it hard to take your medication, attend to your appointments, stick to your diet*	*18*
*Overdose risk*	*A lot of patients with COPD and asthma who are insufflating. That puts them at high risk for overdose and respiratory failure. Physicians are not comfortable prescribing methadone to these patients because they believe that methadone poses a risk for overdose, however, the other alternative is that the patient suffers from respiratory failure*	*5*
	*I don't see them [older adults] as risk takers because they’ve been able to take it [opioids] for so long and survive. If there’s a risk for overdose, I think it has more to do with the components of the drug and its typically fentanyl. If fentanyl wasn’t in the system, then I think they’d be ok because they tend not to use a lot and be greedy. They’re knowledgeable and cautious based on their experience*	*3*
	*A lot of the attention in harm reduction is directed towards those who are injecting drugs, but I think overdose prevention efforts target more people who inject and not as many who snort drugs. However, you can definitely snort and die. A lot of people think snorting is a safer way of using*	*22*
	*I think all of our attention goes to the injectors because they are at higher risk for HIV and hepatitis C even though Hep C can also be transmitted by using straws to snort. I think there’s a lack of program and research focused on that group for that reason*	*1*
	*A patient who is 85 years old has a lot of confidence that they can safely use since they’ve been doing it for so long*	*13*
	*Sometimes the older patients and those who only snort often get overlooked. Maybe they’re not injecting, so they’re not getting the same attention since they don’t go to needle exchange programs and receive Narcan treatment*	*22*
	*I’ll ask if people think it’s safe to do that [using alone] and they’ll say I know my dealer and it’s been the same guy. They have these strategies that they enact that they believe will reduce their risk and, in some cases, may work. In some cases, they’ll test a little bit first and then say, ‘I’ll do more’. Some people will say ‘I tell by the color of it’. More often than not, it’s been I use the same person*	*3*
*SUD treatment and older adults*	*A lot of people think they have to enter a specific treatment program and maybe they are not as familiar with the office-space care*	*17*
	*They probably don’t understand that treatment for OUD is as accessible as it is now. They tend to be very surprised when they find out they can walk out with a prescription for a week or two of suboxone*	*17*
	*Older adults are maybe more wary of treatment because they may have had negative experiences with treatment in the past and may have been stigmatized. They’re maybe not familiar with the new breed of providers who are really trying to make an impact in their community*	*17*
	*Older tropes/patients have had discrimination and negative aspects of disclosing that SUD. They’ve been using for a while but are seeking treatment now because the stigma has been removed*	*19*
	*Older adults are less likely to travel…accessibility is what draws a lot of people in vs them actually having to engage with the healthcare system and I think that’s a big benefit to our older population. Like the convenience of it. I think programs need to be more convenient and flexible*	*1*
	*Because of how complicated their chronic medical conditions are, that makes them difficult to access addiction treatment. It may be hard to leave the house and go to a methadone clinic or do buprenorphine in an office-spaced clinic. They may need post-acute care. Accessing addiction treatment while going through post-acute care can be tricky. We are not just treating addiction, we are also treating other comorbidities, congestive heart failure, chronic kidney disease, post stroke care, traumatic injuries*	*18*
	*The ones we know have been using for quite a while. They’re older and they’ve been able to survive. They tend to figure out other ways to manage their addiction. Very often, drug treatment is part of it, and it is part of their overdose risk, too. If they’re using street drugs and using methadone, there’s an elevated risk of overdose for some folks. As they’re older, their hustling strategies—their ways to raise money for their drug use—is not there. It just gets harder as you are older. They tend to figure out an easier way to use in conjunction with their medically assisted treatment*	*3*
	*If someone has mild to severe cognitive impairment, they may not remember to take buprenorphine or naloxone on a daily basis or they may not remember when they last used. There are some questions there from what should we do from an ethical perspective and operational perspective*	*18*
	*Older patients are usually pretty receptive to treatment. 50/50 between those who want to start suboxone vs those who want to start methadone (what older adults are familiar with). Nearly everyone who I have offered help or treatment to after screening over the age of 50 has been pretty appreciative of that*	*17*
	*The last time I was on a consult service I saw a patient who had a stroke. He was also using opioids and had been using intranasal opioids for a long time. I think our consult service had seen this patient in the past maybe a year ago and had tried to start him on suboxone at that time, but it doesn’t seem like that linkage to care happened. After this stroke, the patient was going to need acute rehab so we were able to restart him on suboxone and connect for him to go on acute rehab on the suboxone. I think this case is similar to most cases since it’s someone who has been using for decades, who was not in for treatment and was open to treatment. It’s someone who we were able to start and link them to care. But I think it’s someone who depending on their degree of recovery from stroke, it may be difficult for them to schedule follow up appointments (securing transportation maybe)*	*18*
	*Nearly everyone who I have offered help or treatment to after screening over the age of 50 has been pretty appreciative of that*	*17*
*Screening*	*So I think a lot of the older patients have had, you know, discrimination or, you know, negative aspects of disclosing that with people. Like, even in my primary care, you know, with new patients, they don't necessarily disclose that on the first visit. Sometimes it takes much longer, especially with methadone, because you can't really track that and like any kind of external, you know, refill history or things like that. So, if they don't tell you, you really don't know*	*19*
	*I asked all my patients very early on, and just my like, a review of their past medical history. I go straight to substances, tobacco, alcohol and everything else. And I asked, you know, if not now, has it ever been a problem? So I do my own personal just like questioning, no formal screening questions. What prompts it…falls and cognition would be my two big ones. And just like any self-care concerns, environment at home, their ability to perform, like, if I have any sense of like, their ability to perform ADLs, whether it's just like, how they're dressed at the appointment, their cleanliness? Just want to make sure that I rule out something like that*	*13*
	*We're trying to build it into our EMR so that… everyone gets at least like a short screener, but we're not there yet. But it is something that is a goal because I do think the providers ask like, oh, do you use substances and stuff? And sometimes that's enough, but sometimes people don't. So they might ask about alcohol and smoking, but they don't necessarily ask about other substances or about, you know, for people who are on chronic opioids, making sure to ask about like any signs that they may be developing that use disorder. So I think that we could do better with like automating that so that people always ask the questions*	*22*
	*There’s some concern that—In older adults, the screening tools that we have can potentially underestimate and overestimate patients having OUD. Some of the diagnostic questions on DSM regard the drug use impairing someone's ability to work or at school. And probably, the older adults is not working anymore, so those questions are hard to apply. On the flip side, they could be showing signs of opioid misuse like they are confused or sedated in some way. And that can be a result of another medical process and be a false positive for opioid use. You can get false positives and false negatives from the screening tools*	*18*
	*I think, in particular, for like patients with chronic pain, I think it's important to ask a little bit about like how the medications might be impacting their life and sort of asking about any impact on behavior or like cravings for or do they feel like they're wanting to use more, like take more than they're prescribed? And I think… a lot of this comes out, you know, there's so much stigma around substance use disorder and especially, I think for patients on chronic opioids, for chronic pain, there's like a lot of fear of getting labeled as someone with a use disorder and then being cut off of all their medication and then they have all this pain*	*18*
	*So I think you have to be really aware of like how we ask the questions and to make sure that patients know that like we ask the questions because we care about them and we want them to do well and not because we're like trying to find, you know what I mean? Like catch somebody who hasn't used disorder and then like ban them from the clinic or something, which unfortunately is kind of what happens sometimes. So I think that there's a way to ask questions without it and making it a collaborative kind of discussion without it being like a—I don't know what's the word I'm looking for—like accusatory kind of questioning*	*18*

### Care seeking

Care providers reported that usually older adults who use NMOs initially presented for a wide array of health concerns other than opioid use. For outreach workers, this included Hepatitis C and HIV services. For primary care providers, it encompassed a wide array of health issues including chronic condition management and acute condition treatment. Emergency medicine physicians reported treating emergent needs such as injury, back pain, and opioid overdose. Those working in acute care settings, pain clinics, hospitals and skilled nursing facilities reported encountering older adults who use NMOs in treating post-surgical recovery, and comorbid health condition complications. Oftentimes, these complications resulted from NMO use.

Most providers reported working with patients/clients to address presenting health concerns first and secondarily, screening for or discussing NMO use. Most health care providers in primary care settings and outreach workers reported subsequently offering treatment for opioid use to these patients and clients with the goal of stopping or reducing NMO use at initial and follow up visits.

### ”Complicated” patients

Health care providers characterized older adults who use NMO as complex patients presenting with multiple needs. Many participants attributed this to comorbidities, which could complicate recovery plans and increase risk of overdose.

Most health care providers described their older adult patients who use NMO as presenting with needs requiring the provider to disentangle issues related to opioid use and use disorder from comorbid physical and mental health conditions. Providers also spoke of dilemmas in treating physical health conditions when the patient is using NMO opioids. The following example, provided by a participant, illustrates this dilemma. An older adult patient who was using NMO was a candidate for a knee replacement. The orthopedic surgeon could not move forward with the knee replacement because they were reluctant to operate on someone using NMO and the patient was unable to stop using NMO due to their pain.

Furthermore, participants conveyed that NMO use may make it difficult for older adults to manage their care for comorbid conditions because opioid use may lead to difficulty in maintaining routines, self-care, and relationships—all of which are typically needed to manage health conditions.

Acute and recovery care for persons who use NMOs that experience a health issue is complicated by opioid use. For example, one participant reported caring for an older adult who used NMOs and experienced a stroke. In addition to arranging for post stroke recovery, the provider had to find a skilled nursing facility that could also offer substance use disorder (SUD) treatment.

Providers also encountered diagnostic difficulties in differentiating symptoms associated with opioid use vs physical and mental health conditions. As some participants pointed out, some NMO use symptoms are also symptoms occasionally associated with health conditions of aging – for example, appearing confused, disheveled, or forgetful. Participants described the “teasing out” of symptoms and conditions as a challenge in working with this population. This “teasing out” was seen as a difficult process by some providers, and they were skeptical that their patients shared their opioid use history completely or accurately.

### A different kind of opioid use disorder?

Primary care medical providers in our study also reported that a significant segment of the older adults who use NMOs they encountered did not meet the Diagnostic and Statistical Manual of Mental Disorders (DSM) diagnosis criteria for opioid use disorder (OUD) in that their current opioid use did not interfere with functionality in terms of serving as a caregiver or maintaining employment.

In contrast, most outreach workers and emergency medicine physicians in our study described the population of older adults who use NMOs as presenting with signs of OUD along with social isolation, extremely low income, and housing instability. Some also noted that there was concurrent alcohol misuse among some older adults who use NMOs.

### Overdose concerns

Most study participants perceived older adults who use NMO as deploying strategic risk reduction behaviors that could protect them from overdose. These included using smaller amounts of opioids less frequently and having a consistent supplier and reliable product.

While acknowledging these older adults exhibit behaviors protective against overdose, most participants perceived older adults who use NMOs at elevated risk of overdose for several reasons. One is the change in the drug supply toward the ubiquitous presence of fentanyl, a strong synthetic opioid. Most noted that older adults who use NMOs began their opioid use decades ago when much less powerful heroin was the dominant substance in unregulated opioid markets. Providers saw the presence of fentanyl as a threat for overdose to older adults who have used NMO for periods of time.

Another threat is the health and cognitive problems associated with increased age and the possibility of overusing because of forgetfulness. The presence of health conditions such as Chronic Obstructive Pulmonary Disease makes opioid use more dangerous because it can cause repressed respiration—a risk for overdose, especially in a population with a preference for insufflation.

One of the perceived overdose risks for older adults was a lack of specific outreach programs for those who snort NMO, as older adults are reported to do. Needle-exchange focused harm reduction and outreach services can often overlook older adults. This may limit older adult’s access to naloxone or knowledge of emerging threats such as the increased presence of Xylazine in the drug supply as these are often distributed through needle-exchange programs.

A perceived risk for overdose among older adults who use NMO is, ironically, their survivorship confidence. Some providers reported cautioning patients on the risks of overdose and hearing back that they “know what they are doing.” However, providers noted that the unregulated drug market has become more dangerous and physical challenges of aging should cause the patient to reassess their risk.

### Substance use disorder (SUD) treatment and older adults

Most of the participants in our study indicated that older adults who use NMOs could benefit from treatment, even if they did not meet the diagnostic criteria for OUD. In their perspective, treatment for NMO use was a means to help these older adults maintain and improve their overall health*.*

Participants reported that older adult patients in their care who use NMO had varied experiences and attitudes towards treatment for substance use. Many had been through treatment in previous decades prior to the availability of medication assisted recovery and had negative and/or unsuccessful experiences. Participants noted that in their experience, older adults who used NMO were surprised when offered treatment through a primary care provider instead of a specialized treatment center. Providers felt that older adults who use NMOs often avoided these centers because of stigma concerns. Many noted that treatment may look different for those who have sustained their NMO use for decades, as opposed to those with recent onset of use.

Participants saw some older adults in substance use treatment as using treatment strategically to maintain their use of NMO, while simultaneously fulfilling their responsibilities and maintaining their current lifestyle. Older adults who use NMO while in treatment were seen as particularly tricky to manage because using methadone along with NMO could increase risk of overdose, especially among older adults with health issues.

According to providers, older adults face age-related barriers to treatment for substance use. Some are logistical issues, such as transportation and travel arrangements, some are health related and specific to their comorbidities. For example, cognitive impairment associated with aging may impose barriers to the provider’s ability to treat the OUD since older adult patients may not be as likely to remember when to take their doses of buprenorphine. Another complication for treatment in this population is the potential need for caregiver involvement. For providers, this necessitates working to communicate the issue and potential treatment options to older adult patient caregivers to gain their support and, in some cases, approval. This is particularly difficult in cases where caregivers do not wish to acknowledge older adults in their care use NMOs.

### Screening

While universal screening for older adult substance use is recommended, [21] not all providers did so. Participants discussed a continuum of screening approaches. This included universal screening, where everyone was screened at every visit and a comprehensive assessment was conducted at first visit; selective screening of those with opioid prescriptions for use of other substances that might interact with opioids such as alcohol; and casual conversations about substance use with the goal of explaining why it is important to know and to encourage disclosure. Medical providers in non-primary settings reported no consistent screening practices and some expressed skepticism about the utility of screening in their context. Outreach and mobile care providers did not conduct screening for substance use because they assumed their patient population use NMOs. Those who screened for NMO use relied on several modalities – Electronic Health Record built-in tools, two question screeners, more comprehensive self-report screening tools, and caregiver consultation.

Participants discussed that screening older adults for NMO use may have multiple benefits in improving the care they could provide. For those who may be prescribed opioids, it can help determine use of substances that might interact with prescribed medications. Another purpose was to detect OUD and initiate conversation on treatment options for those with positive screens.

Participants also discussed how screening and care models can be adapted to accurately reflect older adult characteristics. It was noted that the current DSM OUD criteria is not applicable to many older adults using NMO and may need to be adapted to reflect occupational status more reflective of older adult demographics and other life stage indicators for a more accurate OUD diagnosis in older adults.

## Discussion

The participants in this study offer unique perspectives based on their experiences working with older adults who use NMO. This study has surfaced broad themes for further investigation. As such, these findings are best interpreted to inform further investigation in urban areas with longstanding and entrenched opioid misuse.

Providers in our study described older adults who use NMO as complex patients or clients due to interactions between chronic health conditions and NMO use, which complicates diagnosis and treatment. This suggests that specialized cross-training in addiction medicine for physicians and nurses in geriatric care could enable them to better meet the needs of older adults who use NMO in locations with entrenched opioid use problems. Some study participants have pursued additional certification to better meet the needs of their patients/clients. Increasing health care staff cross-trained in substance use treatment can enable the integration of substance use screening and treatment into emergency, primary, and skilled nursing rehabilitation care settings, where many older adults who use NMO are likely to seek care for comorbid conditions. This strategy can reduce barriers to receiving treatment, such as level of care needed for comorbid conditions, stigma, and travel limitations for older adults. For example, capacity to provide SUD treatment in a skilled nursing facility may enable older adults to receive needed rehabilitation care without interrupting SUD treatment. Cross training in pain treatment modalities for persons who use NMO may also assist providers in addressing conditions which cause pain and, consequently, lead to NMO use. For example, training in the dispensing of buprenorphine or buprenorphine-naloxone combinations for chronic pain management in persons with opioid dependence are potential treatment avenues.

More research is needed on primary care and geriatric provider attitudes toward cross-training, barriers to training, potential models for training delivery, and desired topics for cross-training. Potential topics for cross training research include strategies for pain management in persons who use opioids, comorbidity management strategies for persons who use NMO, strategies for engagement of skilled nursing and assisted living facilities for treatment care and support, and best practices for screening substance use among older adults.

While NIDA recommends universal screening for OUD, participants in our study reported varied practices and attitudes towards screening despite working in environments with known populations of older adults who use NMOs. This suggests more work is needed to uncover nuances in screening needs among older adults. Participants made several suggestions for the revision of standard screening content to better reflect the lifestyle situations seen among older adults who use NMO. Input from multiple perspectives, including providers and caregivers working with older adults who use NMO, as well as older adults with lived experience is needed to develop useful and effective screening tools and protocols for older adults. Broadening the contexts in which OUD screening for older adults occurs is another potential strategy to increase opportunities for referral to evidence-based treatment and identify supports for persons who use NMO. Investigation into providers’ attitudes toward screening, ideas for screening content and implementation, and exploration of the logistical barriers which inhibit screening implementation in a variety of settings (social service sites, rehabilitation facilities, primary care, home health care, emergency department and pain management clinics) is needed.

While the larger discussion of NMO use generally focuses on severe OUD, participants in our study discussed a continuum of opioid use patterns among older adults that is generally not addressed. Due to co-morbid health conditions, the effect of aging on metabolism, and long-established use patterns, older adults may experience negative outcomes at lower use levels and less severe opioid use patterns. Understanding the use points at which negative outcomes occur for older adults is important to developing screening tools, counseling approaches and treatment access pathways for older adult who use NMOs.

Some participants reported that older adults who use NMOs in their care continued to use NMO while participating in medication assisted recovery. Providers believe that these patients/clients’ goal for this was to manage their opioid dependence in the long term. While this strategy presents some risk of overdose and has implications for treatment of medical conditions, some older adults have leveraged this to effectively manage their use. More research is needed on provider awareness and attitudes around the strategic use of treatment by older adults.

Some study participants identified barriers to treatment for older adults who use NMO and found that offering medication(s) for opioid use disorder in a primary care setting could overcome stigma against attending treatment in a stand-alone treatment center while improving the quality of comorbid condition management. However, providers noted that some older adults who use NMOs in their care had previously participated in treatment and were not aware of the newer options for treatment in a primary care setting. This suggests research on the awareness of older adults who use NMOs on current treatment options and development of better ways to communicate these new options to older adults. Harm reduction outreach may be one avenue to educate older adults, however because of stigma and a heavy focus on needle exchanges, these efforts may miss older adults who are secretly using NMO. Distribution of educational materials on medication assisted recovery and its availability in primary care and ED settings should be expanded to contexts where services are provided to older adults such as senior centers, congregate meal locations, and senior housing settings.

One barrier to treatment specific to older adults is the cognitive decline which accompanies aging. This may prevent some older adults from benefiting from treatment and may require providers to develop assessment tools to determine the best course of treatment for these situations. More research is needed to assess the extent to which cognitive decline may be a factor in treatment participation and the development of strategies for alternative approaches for these individuals.

This study highlights need for more research on harm reduction services beyond needle exchanges and outreach strategies that can reach the older adults who use NMOs, particularly for those who perceive strong stigma towards identifying as a person who uses NMO and uses in a clandestine manner. Harm reduction professionals in our study reported that their ability to reach older adults who use NMOs is limited given current operating models. More research with this population is needed to explore services that are needed and desired by older adults using NMO, services that appeal to this population, and models of service delivery.

## Conclusions

Much remains to be learned about the health and outreach encounters of older adults who use NMO. This qualitative study found that health care providers see treatment of physical health co-morbidities among older adults who use NMO as complex. A next step could be to assess providers’ needs for supports to better serve this population. Additional areas for further exploration following this initial study include gathering input from health care and outreach providers as well as older adults with lived experience with substance use to inform: development/adaptation of substance use screening protocols geared to older adults; strategies to communicate advancements in OUD treatment to older adults who may have had negative treatment experiences in the past, and development of harm reduction outreach models geared older adults who use NMO. The context of the study setting may make our findings more applicable to other large urban settings with significant and entrenched opioid overdose problems.

## Limitations

Study limitations include the absence of perspectives from older adults using NMO. The scope of this study is on the perspectives of providers who encounter older adults using NMO. It is possible that these providers see older adults using NMO, but the use unknown to providers. Therefore, viewpoints of older adults who use NMO would help to expand the topics covered. The perspectives of older adults themselves is hoped to be explored in consequent studies. The focus on areas with high NMO usage may make this study difficult to translate to communities with differing rates of NMO usage. The study’s Chicago focus requires consideration when applying study findings to other regions that may have different histories of opioid use, demographics, and other factors which could influence the nature of NMO use. Furthermore, as with any qualitative study, there is the possibility of personal biases influencing the perspectives of participants.

## Data Availability

Data can be found in the subsequent Tables 1 and 2. For the complete data sets, including transcripts of interviews, please contact Dr. Maryann Mason of the Buehler Center.
